# Quantification of arterial hemodynamics in steno-occlusive disease using time-resolved MRI-based angiography

**DOI:** 10.1177/0271678X261500385

**Published:** 2026-10-20

**Authors:** Quinten Deckers, Simone M Uniken Venema, Kees P Braun, Albert van der Zwan, Tristan PC van Doormaal, Pieter T Deckers, Jeroen CW Siero, Alex A Bhogal

**Affiliations:** 1 Translational Neuroimaging Group, Center for Image Sciences, UMC Utrecht, Netherlands; 2 Department of Radiology and Nuclear Medicine, UMC Utrecht, Netherlands; 3 Brain Center, Department of Neurology and Neurosurgery, UMC Utrecht, Netherlands; 4 Precision Imaging Group, Center for Image Sciences, UMC Utrecht, Netherlands

**Keywords:** 4D-MRA, arterial transit time, ASL-MRI, steno-occlusive disease, vascular steal

## Abstract

**Background:**

Intracranial steno-occlusive disease (SOD) assessment benefits from hemodynamic imaging, but comprehensive evaluation often relies on contrast- or radiation-based techniques. Arterial spin labeling (ASL) provides a non-invasive alternative for quantifying tissue-level perfusion and cerebrovascular reactivity, yet does not capture upstream arterial flow dynamics. As a result, non-invasive assessment of macrovascular hemodynamics for SOD remains limited. This study therefore investigates the relationship between quantitative 4D-MRA derived parameters and established ASL-derived metrics to provide insight into the relationship between macrovascular arterial transit and downstream tissue-level perfusion, which helps to define the potential role of 4D-MRA-derived parameters in future cerebrovascular assessments.

**Methods:**

Twelve SOD patients (7 women; age 42.3 ± 25.8 years) underwent multi-delay ASL and 4D-MRA before and after acetazolamide. Cerebrovascular reactivity (CVR), arterial transit time (ATT), macrovascular ATT (mATT), and labeled blood volume (LBV) were quantified. Associations and vasodilatory responses were assessed using linear mixed-effects models.

**Results:**

At baseline, mATT correlated with ATT (β = 0.66 ± 0.08, *p* < 0.001). Both decreased following acetazolamide (mATT: 1.07 ± 0.03 s to 1.01 ± 0.03 s, *p* = 0.029; ATT: 1.63 ± 0.07 s to 1.40 ± 0.07 s, *p* < 0.001). However, changes in mATT and ATT were not associated with CVR. In contrast, CVR was positively associated with ΔLBV (β = 8.84, SE = 2.43, *p* = 0.01). Case analyses further demonstrated delayed inflow and vascular steal at the level of individual arteries.

**Conclusion:**

Quantitative 4D-MRA provides complementary macrovascular information to ASL in SOD. ΔLBV more consistently reflects cerebrovascular reactivity than transit-based metrics and is sensitive to artery-level delayed inflow and vascular steal.

## Introduction

In intracranial steno-occlusive disease (SOD) like moyamoya disease, hemodynamic information can support diagnosis, treatment planning, and follow-up.^1,2^ For example, stenosis severity derived from structural MRI does not provide information on tissue-level cerebral blood flow (CBF) or autoregulatory compensation.^3,4^ In our current clinical MRI protocols for SOD patients,^5^ multi-delay arterial spin labeling (md-ASL) is used to quantify CBF, arterial transit time (ATT) and cerebrovascular reactivity (CVR).^5–9^ When deciding on surgical revascularization, these data are considered alongside clinical parameters and digital subtraction angiography (DSA), which is done to assess stenotic severity and collateral pathways.^10,11^ DSA is invasive and, while acceptable for initial diagnosis, unsuitable for serial monitoring.^1,12^ For optimal patient selection, and to facilitate treatment monitoring and follow-up, a non-invasive hemodynamic workup would be ideal. This highlights a clinical need for radiation- and contrast-free MRI methods that deliver large-vessel hemodynamic information.

One candidate is ASL-based time-resolved magnetic resonance angiography (4D-MRA). 4D-MRA uses magnetically labeled blood to capture arterial flow dynamics across multiple inflow times. By fitting an appropriate signal model, macrovascular arterial transit time (mATT) can be quantified, which provides information that may reflect collateral routing and proximal flow impairment.^13^ By introducing a vasoactive stimulus such as acetazolamide, the quantitative 4D-MRA protocol can also inform on CVR responses via changes in labeled blood volume (LBV), an image-derived measure representing the total volume of voxels occupied by labeled arterial blood. An increase in blood flow velocity is expected to increase LBV because (1) more labeled spins pass through the labeling slab and (2) these spins travel further along the vascular tree between labeling and image acquisition. Under this formulation, LBV may serve as an arterial surrogate for the CVR response and may provide a means of identifying vascular steal phenomena at the level of the large intracranial vessels. At the tissue level, vascular steal is characterized by a paradoxical reduction in blood flow within compromised regions following a vasodilatory challenge. This may occur when in territories with exhausted or impaired vasodilatory reserve compete with limited blood supply during a global vasodilatory challenge.^14,15^ In conventional perfusion imaging, this manifests as a reduction in CBF, expressed in ml/min/100 g.^15^ In the upstream feeding arteries, we hypothesize that the same phenomenon may be reflected by a reduction in flow velocity and a corresponding prolongation of arterial arrival times. Under these conditions, labeled blood may not propagate through and fill the vascular tree to the same extent as under baseline flow conditions.

To determine whether quantitative 4D-MRA and multi-delay arterial spin labeling (ASL) provide concordant hemodynamic information, we compared 4D-MRA–derived mATT and LBV with their corresponding perfusion-based metrics in a clinical population with steno-occlusive disease. We hypothesized that mATT would be shorter than ASL-derived ATT, reflecting proximal rather than tissue-level arrival, and that vasodilation would increase CBF, shorten both transit-time parameters, and increase LBV through deeper penetration of labeled blood into the arterial tree. We additionally examined whether regions affected by vascular steal deviated from these expected response patterns.^16,17^

## Materials and methods

This single-center interventional study was performed at the University Medical Center Utrecht (UMCU; The Netherlands). The local Medical Ethical Review Committee NedMec declared that the Medical Research Involving Human Subjects Act (WMO) did not apply (trial nr. 21-406) and approved the study accordingly. All participants or their legal representative provided written informed consent to use their data. As this study is a proof-of-concept, no sample size calculation was performed.

### Study participants

Participants were included between July 2024 and March 2025 if they had confirmed SOD and a clinical indication for hemodynamic MRI as determined by the treating physician. All participants underwent MRI including ASL-based 4D-MRA and multi-delay ASL (md-ASL) before and after intravenous administration of acetazolamide within a single imaging session. Clinical indications included: evaluation of suitability for bypass surgery, assessment of cerebrovascular reactivity in the setting of new ischemic symptoms, or postoperative evaluation following neurosurgical intervention. Both pediatric and adult participants were eligible, irrespective of prior extracranial–intracranial bypass surgery. Only participants with complete pre- and post-acetazolamide ASL-based 4D-MRA and md-ASL datasets suitable for quantitative analysis were included. Participants were excluded if imaging was incomplete, degraded by severe motion artifacts, or if contraindications to MRI or acetazolamide were present (see “Participant Characteristics” in Results). Data from four participants were previously reported to introduce the mATT estimation model^13^; however, the present study compares 4D-MRA–derived mATT and LBV with corresponding ASL-derived perfusion metrics to determine whether both techniques provide concordant hemodynamic information.

### MRI protocol and data acquisition

All participants were scanned on a 3T scanner (Philips Healthcare, Best, the Netherlands) using a 32-channel receive coil (Invivo, Gainesville, USA). The clinical protocol^18^ contained a combination of standard structural imaging, md-ASL, and the pseudo-continuous arterial spin labeling, combined with Keyhole and View-sharing scan (4D-PACK),^19^ which we refer to herein as 4D-MRA. The total scan duration was 45 min.

A dynamic pseudo-Continuous Arterial Spin Labeling (pCASL) sequence with a variable repetition time (vTR-ASL) and optimized background suppression was used for multi-delay ASL measurements. vTR-ASL enables flexible label durations and post-labeling delays, making it an efficient method for CBF and ATT assessment.^13^ The ASL labeling plane was positioned manually to be perpendicular to the internal carotid and vertebral arteries. Arterial signal suppression was achieved using improved motion-sensitized driven-equilibrium with a TE of 16 ms and velocity encoding of 5 cm/s in three directions.^20^ The pCASL sequence contained a 3D GRASE readout, 10 label durations (300–2000 ms), and 10 post-labeling delays (200–3000 ms). Additional settings were turbo spin echo factor = 15, EPI factor = 15, SENSE factor = 2.5 × 1, half scan factor = 1 × 0.8 (right-left, feet-head direction), TR/TE range 1650–6150 ms/15 ms, flip angles = 90°/180°, voxel size = 4 × 4 × 8 mm^3^, field of view = 256 × 240 × 120 mm^3^, scan time = 2 min 54 s. The unique label duration and post-label delay combinations resulted in 10 saturation delays with a constant step size (500–5000 ms). A magnetization (M0) calibration image for CBF quantification was acquired with a 3000 ms TR. CBF and ATT quantification followed the Buxton kinetic model with nonlinear fitting,^21^ using fixed parameters: labeling efficiency = 0.85, background suppression efficiency = 0.8, tissue T1 = 1.5 s, arterial blood T1 = 1.66 s, and tissue-blood partition coefficient = 0.9.

A vendor-specific (Philips) 4D ASL-based MRA sequence was used that incorporates “keyhole” and “view-sharing” techniques as part of the 4D-PACK implementation for scan acceleration.^19^ Our parameters were as follows: 4D-MRA using a 3D GRE readout, 8 time-points at 100, 200, 400, 600, 800, 1200, 1600, and 2200 ms (label durations), 75% keyhole central size, turbo factor = 60, SENSE factor = 3 × 15 (right-left, feet-head direction), half scan (partial Fourier) factor = 0.8 × 0.8 (right-left, feet-head direction), TR/TE/flip angle = 6 ms/1.97 ms/11 degrees, acquired voxel size = 1 × 1.4 × 1.6 mm^3^, reconstructed voxel size = 0.6 × 0.6 × 0.8 mm^3^, field-of-view = 200 × 200 × 120 mm^3^, scan time = 6 min 16 s). Both the 4D-PACK and vTR-ASL sequences are part of Philips’ Advanced ASL Extension patch.

After an intravenous injection of acetazolamide (20 mg/kg with a maximum of 1 g, dissolved in 30cc 0.9%NaCl, flow rate of 0.3 cc/s), a second multi-delay ASL was acquired, as well as a second 4D ASL-based MRA. The post-acetazolamide 4D-MRA and md-ASL scans were acquired 9 ± 2 min and 16 ± 2 min after injection, respectively.

### Image pre-processing and analysis

Multi-delay ASL data were processed using an in-house developed pipeline^22^ to generate pre- and post-acetazolamide CBF and ATT, using FSL BASIL for both the pre-acetazolamide and the post-acetazolamide ASL scan. CVR maps were calculated by subtracting pre-acetazolamide CBF maps from post-acetazolamide CBF maps (using the 2nd to the 5th post-label delay images). As such, ASL-CVR was defined as the absolute CBF change in ml/100 gr/min (= post-ACZ—baseline) and thus an increase in CBF from the baseline to the post-ACZ scan results in a positive ASL-CVR.

For an elaborate description of mATT fitting method, see Bhogal et al.^13^ Briefly, mATT was estimated using a piecewise saturation model, where an initial linear phase represents the arrival time of labeled spins, followed by a nonlinear saturation function describing signal increase. The model’s breakpoint indicates when the inflow signal surpasses the detection threshold, marking label-bolus arrival. More distal arterial voxels required longer label durations before reaching this threshold. Because 4D-MRA confines labeled spins to the arterial compartment, total labeled blood volume (LBV) and its acetazolamide-induced change (ΔLBV) were calculated as arterial markers of cerebrovascular reactivity.

For each participant, all derived parameter maps (CBF, CVR, (D)ATT, (D)mATT, (D)LBV) were parcellated into 91 (sub)cortical regions using the native-transformed Harvard-Oxford atlas.^23^ The Harvard-Oxford atlas was selected to provide finer regional parcellation and improve sensitivity for detecting localized perfusion abnormalities that may be obscured when using larger vascular territory-based atlases. This is particularly relevant in patients with SOD, in whom collateral recruitment through secondary pathways, including leptomeningeal and ophthalmic arteries, may shift perfusion border zones away from their conventionally defined anatomical locations, thereby favoring a finer regional parcellation.^24,25^

To perform the regional analysis in native image space, the first raw label/control image was registered to MNI space using linear and non-linear transformations implemented in FSL.^26,27^ The corresponding inverse linear transform and inverse warp field were then calculated and applied to the Harvard-Oxford atlas, thereby transforming the atlas from MNI space to each participant’s native space. The native-space atlas was subsequently overlaid on the derived parameter maps, and the mean value within each atlas region was calculated. For the vessel-based mATT and LBV maps, regional assignment was performed voxelwise based on spatial overlap with the native-space Harvard-Oxford atlas. Each voxel containing a valid vessel estimate was assigned to the atlas region in which it was located, and regional means were calculated from all valid vessel voxels within that region. Consequently, larger arteries extending across multiple atlas regions contributed voxels to each region according to their spatial location. Background voxels and voxels outside the vessel maps with zero values were excluded from the regional averaging. The resulting regional values were used for subsequent statistical analyses. The full preprocessing workflow is illustrated in Figure 1(a) to (d).

**Figure 1.**
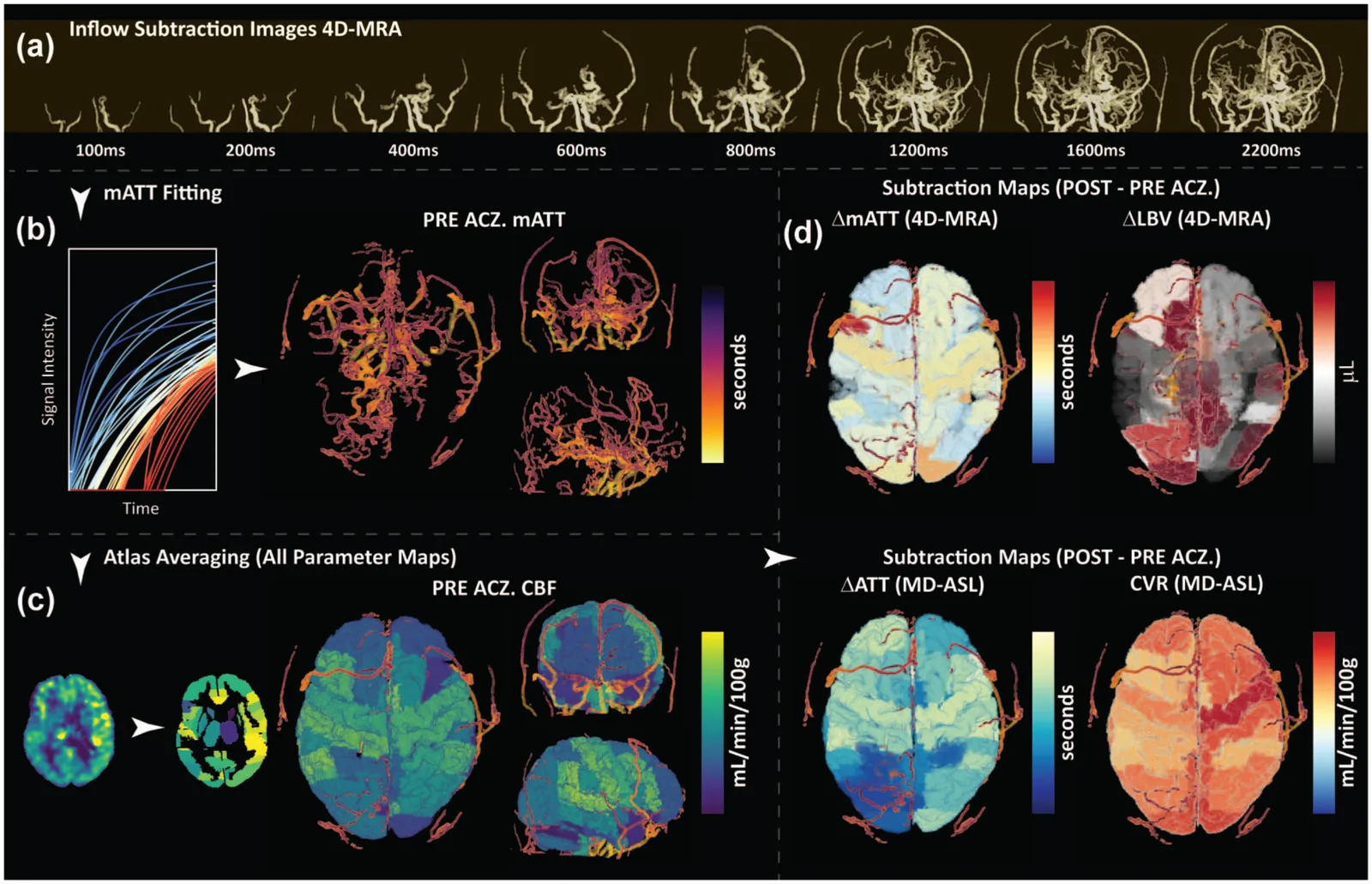
Preprocessing overview; (a) Time-resolved inflow subtraction images from 4D-MRA illustrating progressive arterial filling; (b) Pre-acetazolamide mATT maps generated by fitting a piecewise saturation model to arterial voxels; (c) Atlas-based regional averaging of parameter maps, shown here for pre-acetazolamide CBF, and a 3D render of cortices of these maps with the macrovascular superimposed; (d) Post–pre acetazolamide subtraction maps depicting vasodilatory changes in 4D-MRA–derived mATT and ΔLBV, alongside corresponding md-ASL derived ATT and CVR, with CVR defined as the absolute change in CBF, superimposed on a 3D render of the cortices of the parameter-based map. 4D-MRA: 4-dimensional magnetic resonance angiography; (m)ATT: (macrovascular) arterial transit time; CBF: cerebral blood flow; LBV: labeled blood volume; CVR: cerebrovascular reactivity; ACZ: acetazolamide.

### Statistical analysis

Linear mixed-effects (LME) models were used to assess associations between md-ASL and 4D-MRA parameters. We accounted for within-subject and within-region variability, with subjects and regions included as random effects. Age and sex were initially included as covariates to account for potential confounding. However, as these did not provide any meaningful contribution to the model, they were omitted from the final analyses to preserve statistical power and avoid overfitting. Model selection was guided by Akaike Information Criterion (AIC) to minimize bias in model selection (Table S1).

Three main analyses were performed in R (RStudio) using the lmer function^28^:

1. We assessed the correlation between mATT and ATT under the hypothesis that these correlate positively. We also expected a slight offset, as mATT measures upstream microvasculature compared to the tissue-level.
2. CVR was correlated with D(m)ATT to test the hypothesis that CVR varies inversely with arrival time as a result of increased perfusion after vasodilation.
3. CVR was correlated with DLBV to test the hypothesis that these metrics are positively correlated.

## Results

### Participant characteristics

A total of 18 eligible participants underwent imaging. Data from one participant was excluded because they did not provide consent, leaving 17 participants with a combined total of 34 pre- and post-acetazolamide 4D-MRA acquisitions. Of these acquisitions, 30 were of sufficient quality for analysis, corresponding to a scan-level technical success rate of 88%. As the analyses required paired pre- and post-acetazolamide measurements for both imaging modalities, four participants were excluded because one of their 4D-MRA acquisitions did not meet the required quality criteria due to imaging artifacts. One additional participant was excluded for the same reason in the md-ASL data. The final sample therefore consisted of 12 participants with SOD (seven women; mean age, 42.3 ± 25.8 years; Table 1). SOD was attributed to moyamoya disease (*n* = 7), arterial occlusion (*n* = 3), or atherosclerotic stenosis (*n* = 1); in one participant, the diagnosis remained uncertain, with possible moyamoya disease (Figure 2).

**Table 1.** Additional participant information.

Participant number	Age (y)	Sex	Affected hemisphere (l/r)	Previous surgery*	Diagnosis	Diagnostic modality	MDT assessed
1	46	M	Unilateral (l)	No	M1 stenosis left (atherosclerosis)	DSA & MRA	Yes
2	17	F	Bilateral	No	Unilateral occlusion ICA top, MCA/ACA/PCA left. Right irregular ICA and occlusion ACA	DSA & MRA	Yes
3	55	F	Bilateral	No	Paresis l/r with suspicion of MMD	DSA & MRA	Yes
5	60	M	Unilateral (r)	Yes (r)	TIAs right after EC-IC bypass right, ICA occlusion right	DSA & MRA	Yes
6	42	M	Bilateral	No	New infarcts, known MMD	DSA & MRA	Yes
7	45	M	Bilateral	No	Bilateral MMD	DSA & MRA	Yes
8	66	M	Bilateral	Yes (l)	Status post EC-IC bypass left, now possible TIAs right in MMD; bilateral carotid terminus occlusion with suspicion of MMD	DSA & MRA	Yes
9	16	F	Unilateral (l)	No	Unilateral occlusive arteriopathy left (moyamoya pattern) with migraines	MRA**	No**
11	17	F	Unilateral (l)	Yes (l)	Unilateral moyamoya syndrome, ICA/MCA/PCA left with collaterals, with a capillary hemangioma	DSA & MRA	Yes
14	63	F	Bilateral	No	Bilateral ICA occlusion	DSA & MRA	Yes
16	34	F	Unilateral (l)	No	Stenosis MCA left, possible MMD	DSA & MRA	Yes
17	47	F	Unilateral (l)	No	Stenosis MCA left, possible MMD or atherosclerosis	DSA & MRA	Yes

TIA: Transient Ischemic Attack; MMD: Moya-moya Disease; ICA: Internal Carotid Artery; MCA: Middle Cerebral Artery; ACA: Anterior Cerebral Artery; PCA: Posterior Cerebral Artery; EC-IC: Extracranial-intracranial; DSA: Digital subtraction angiography; MRA: Magnetic Resonance Angiography.

* Surgery refers to the (l/r = left/right) hemisphere combined bypass. **This pediatric participant only received an MRI and was discussed between a pediatric neurologist and vascular neurosurgeon.

**Figure 2.**
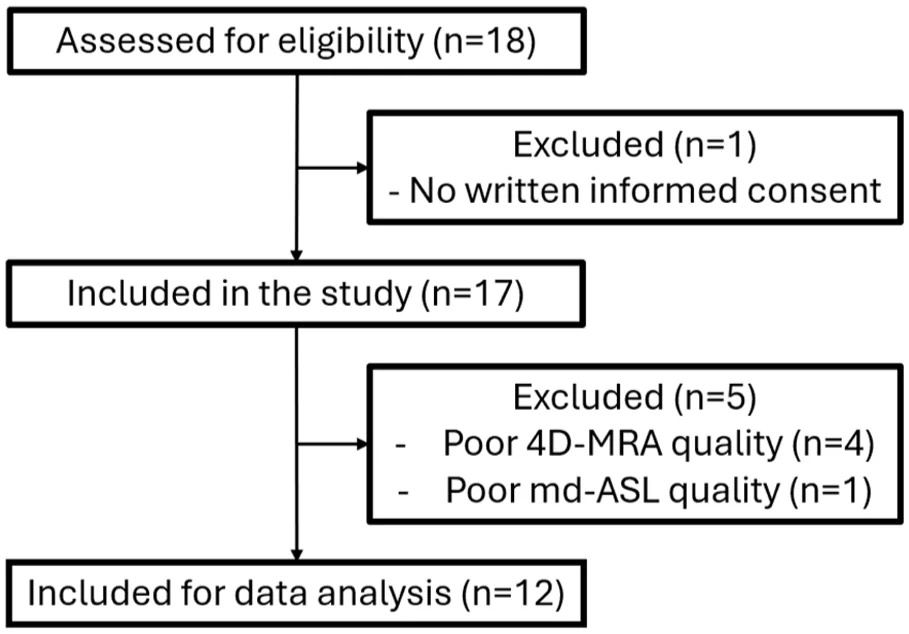
Flowchart shows inclusion for eligible participants with steno-occlusive disease. Md-ASL: multi-delay arterial spin labeling; 4D-MRA: 4-dimensional magnetic resonance angiography.

### Analysis 1: ATT versus mATT

ATT and mATT were positively associated both before (β = 0.66, SE = 0.08, 95% CI: 0.51 to 0.80, *t* = 8.7, *p* < 0.001), and after acetazolamide (β = 0.77, SE = 0.10, 95% CI: 0.58 to 0.96, *t* = 7.8, *p* < 0.001; Figure 3(a)). The best-fitting model included random slopes for subjects and regions, reflecting intersubject heterogeneity and regional vascular differences. As mATT increased, pre- and post-acetazolamide ATT values converged. Significantly different intercepts (pre: β = 0.87, SE = 0.09, 95% CI: 0.68 to 1.05, *p* < 0.001; post: β = 0.56, SE = 0.11, 95% CI: 0.35 to 0.77, *p* < 0.001, difference: *p* < 0.001) indicated a systematic offset between ATT and mATT.

**Figure 3.**
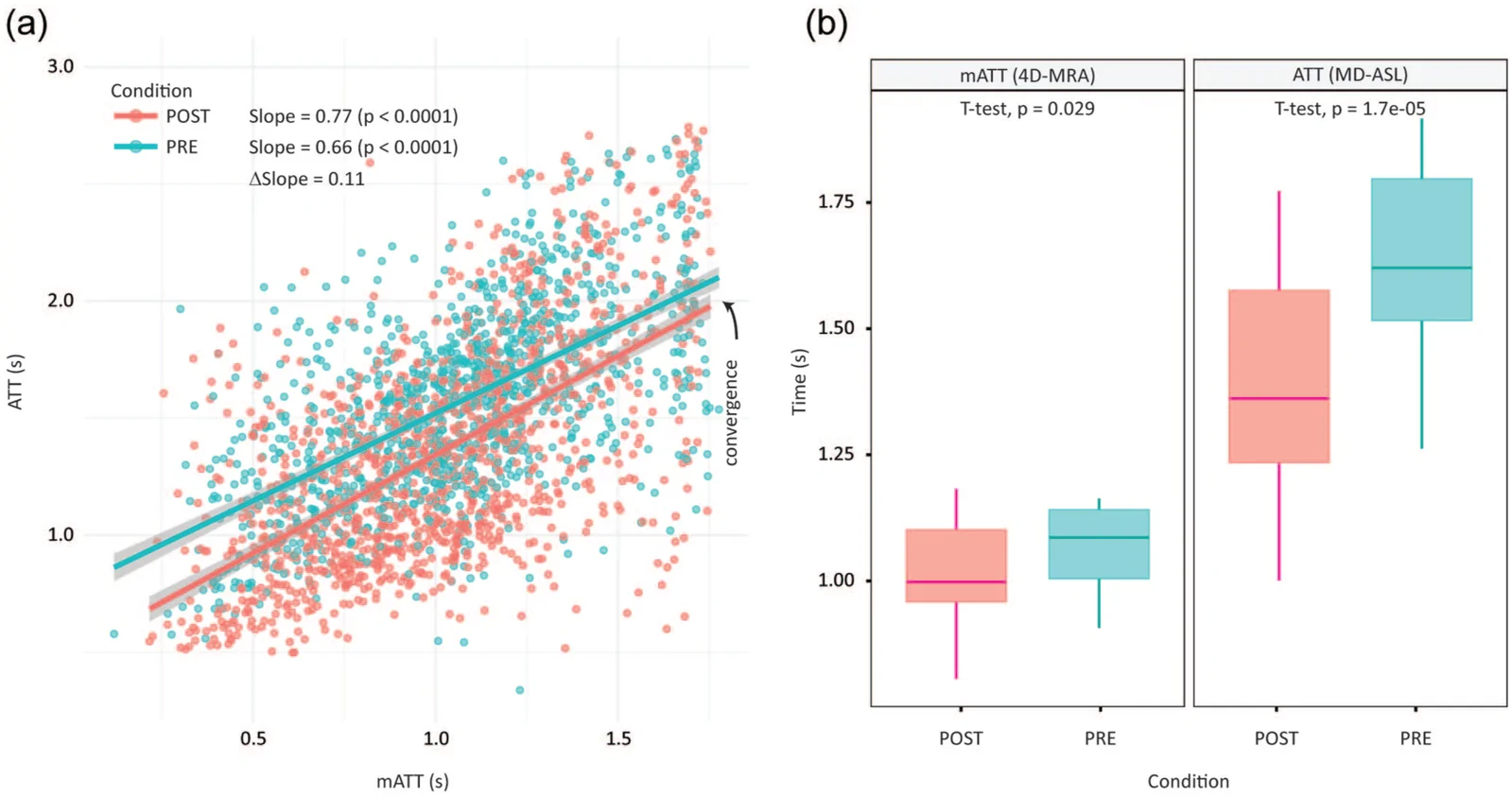
(a) Linear mixed-effects model comparing ATT and mATT pre- (blue) and post- (red) acetazolamide. (b) The effect of acetazolamide on both ATT and mATT reveal a shorter transit time in the post- condition (red) compared to the pre-acetazolamide condition (blue). As noted by the black arrow, the (m)ATT relationship converged at long transit times. (m)ATT = (macrovascular) arterial transit time.

Furthermore, both mATT (pre: 1.07 ± 0.03 s; post: 1.01 ± 0.03 s; *p* = 0.03) and ATT (pre: 1.63 ± 0.07 s; post: 1.40 ± 0.07 s; *p* < 0.001) significantly decreased after acetazolamide, confirming the expected vasodilatory effect (Figure 3(b)). The reduction was larger for ATT than mATT.

### Analysis 2: CVR versus ∆(m)ATT

CVR showed no significant association with ∆ATT (β = 17.2, SE = 8.4, 95% CI: −0.7 to 35.1, *p* = 0.06) or ∆mATT (β = 0.60, SE = 0.44, 95% CI: −0.30 to 1.51, *p* = 0.20). The ∆ATT model explained 73% of the variance, though only 5.5% was attributed to ∆ATT itself, indicating strong subject and regional variability. Participants with higher CVR generally showed lower or negative slopes (Figure 4(a) and (b)). On average, CVR was positive (95% CI: 13.4–14.9 ml/100 gr/min), while ∆ATT was negative (95% CI: −0.21 to −0.19 s), consistent with increased flow and reduced transit time after vasodilation. For ∆mATT, the model explained 56% of the variance (*R*^2^ < 1%), with a similar pattern: positive CVR (14.2 ml/100 gr/min, 95% CI: 13.4–14.9) and negative ∆mATT (95% CI: −0.085 s to −0.060 s). No significant relations were found when comparing CVR with baseline (m)ATT (ATT: *p* = 0.27, mATT: *p* = 0.91, Figure S2).

**Figure 4.**
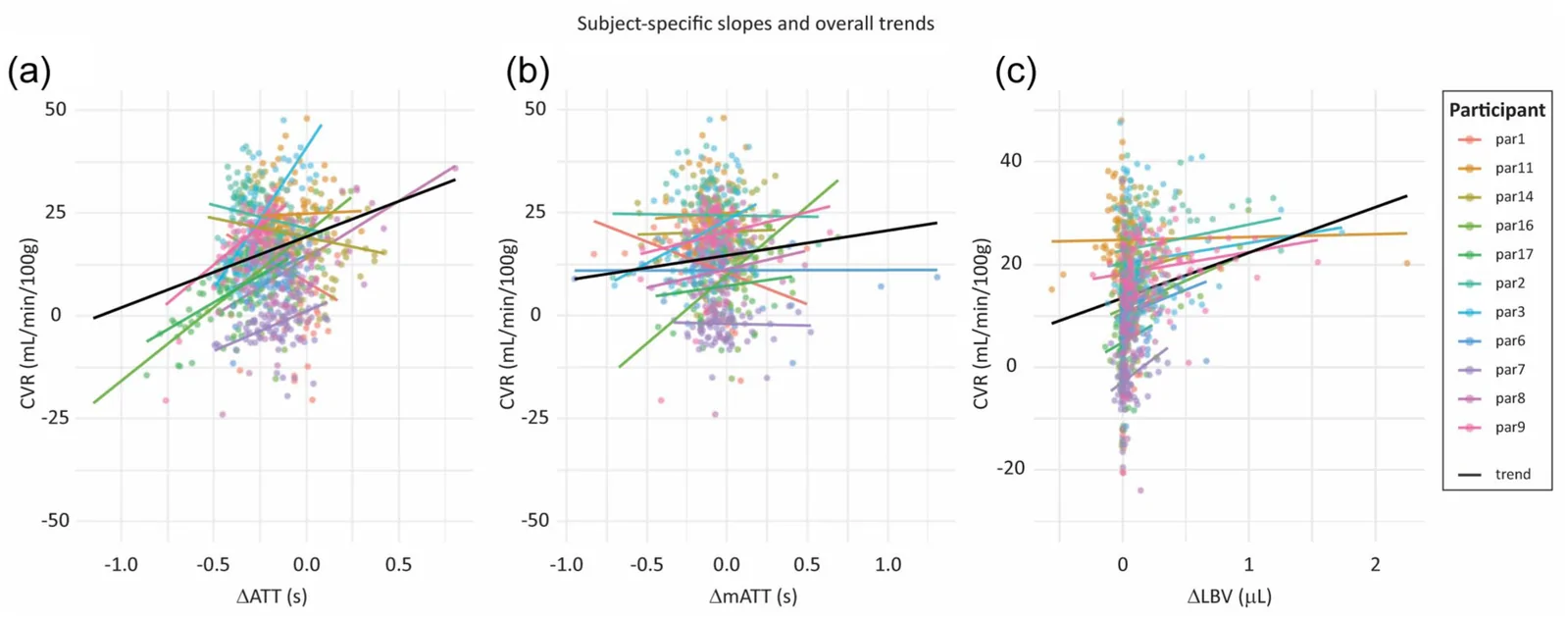
Subject-specific relationships from linear mixed-effects models between CVR and (a) ∆ATT from ASL, (b) ∆mATT from 4D-MRA, and (c) ∆LBV from 4D-MRA. Colored lines represent individual subject slopes with ROI-level data points; group-level trend is shown in black. 4D-MRA: 4-dimensional magnetic resonance angiography; ∆: post—pre acetazolamide; (m)ATT: (macrovascular) arterial transit time; LBV: labeled blood volume; CVR: cerebrovascular reactivity (=∆CBF (cerebral blood flow)).

When dividing unilateral cases (*n* = 6) into affected and unaffected sides, no negative association was detected on either side (Figure S3).

### Analysis 3: CVR versus ∆LBV

CVR and ∆LBV showed a positive correlation (β = 8.84, SE = 2.43, 95% CI: 4.1 to 13.6, *p* = 0.01). Mean CVR was positive (14.2 ml/100 gr/min, 95% CI: 13.4–14.9), and the average LBV increased by 0.15 mL (95% CI: 0.06–0.24) after acetazolamide. All but one subjects showed positive slopes (Figure 4(c); sub11).

Some findings did not align with our original hypotheses. These are presented in short case reports below.

#### Case 1

Negative CVR and ΔLBV (sub17): A 47-year-old woman with left M1 stenosis showed left-sided regions of low to negative CVR and ΔLBV following acetazolamide, indicating reduced perfusion and limited delivery of labeled blood after acetazolamide, compared to the pre-acetazolamide values. Correspondingly, vessels in the affected hemisphere exhibited prolonged mATT, whereas the contralateral hemisphere showed the expected mATT shortening with increased CVR and ∆LBV during vasodilation (Figure 5(a)–(c)). This case illustrates how quantitative 4D-MRA can directly visualize arterial vascular steal at the level of individual arteries, with acetazolamide inducing a negative rather than the expected positive CVR response.

**Figure 5.**
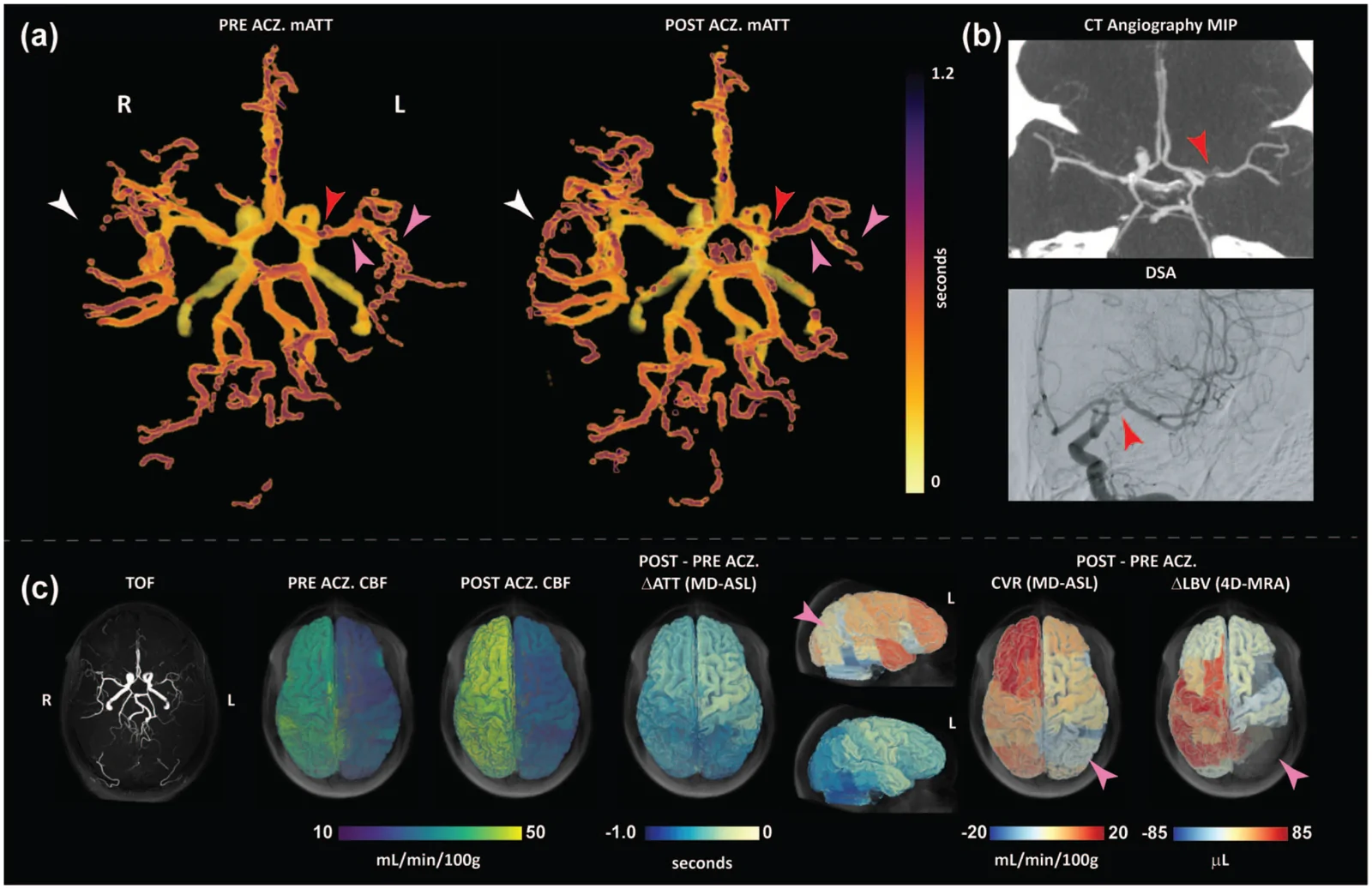
Vascular steal in a 47-year-old woman with left M1 stenosis (red arrows); (a) Pre- and post-acetazolamide mATT maps demonstrate left-sided prolongation of mATT with concomitant decreases in LBV, consistent with vascular steal (pink arrows). In the contralateral hemisphere, mATT shortens with increased LBV, as expected during vasodilation (white arrows); (b) Maximum-intensity-projection CT angiography and digital subtraction angiography (DSA) of the circle of Willis confirm the left M1 stenosis (DSA: zoomed-in AP image with contrast in the left internal carotid artery (DSA: zoomed-in AP image with contrast in the left internal carotid artery); (c) Time-of-flight MR angiography, CBF, ΔATT, CVR, and ΔLBV maps projected onto a cortical volume rendering using the SeeVieweR toolbox^29^ further illustrate vascular steal in the affected territory (pink arrows). (m)ATT: (macrovascular) arterial transit time; CBF: cerebral blood flow; LBV: labeled blood volume; CVR: cerebrovascular reactivity; ACZ: acetazolamide; DSA: digital subtraction angiography; MIP: maximum intensity projection.

#### Case 2

Elevated CBF with prolonged ATT (sub16): A 34-year-old woman with left M1 stenosis showed elevated regional CBF in the affected hemisphere despite prolonged ATT (Figure 6(a)). Voxel-wise CBF hyperintensities colocalized with arteries exhibiting prolonged mATT on 4D-MRA (Figure 6(b)), suggesting overestimation of regional CBF due to intravascular signal contamination, also known as arterial transit artifacts, despite the use of md-ASL. Slow arterial inflow was likely compensated by external–internal collateral pathways observed on the DSA (Figure 6(c)). This case illustrates how quantitative 4D-MRA provides complementary information that aids interpretation of complex hemodynamic patterns.

**Figure 6.**
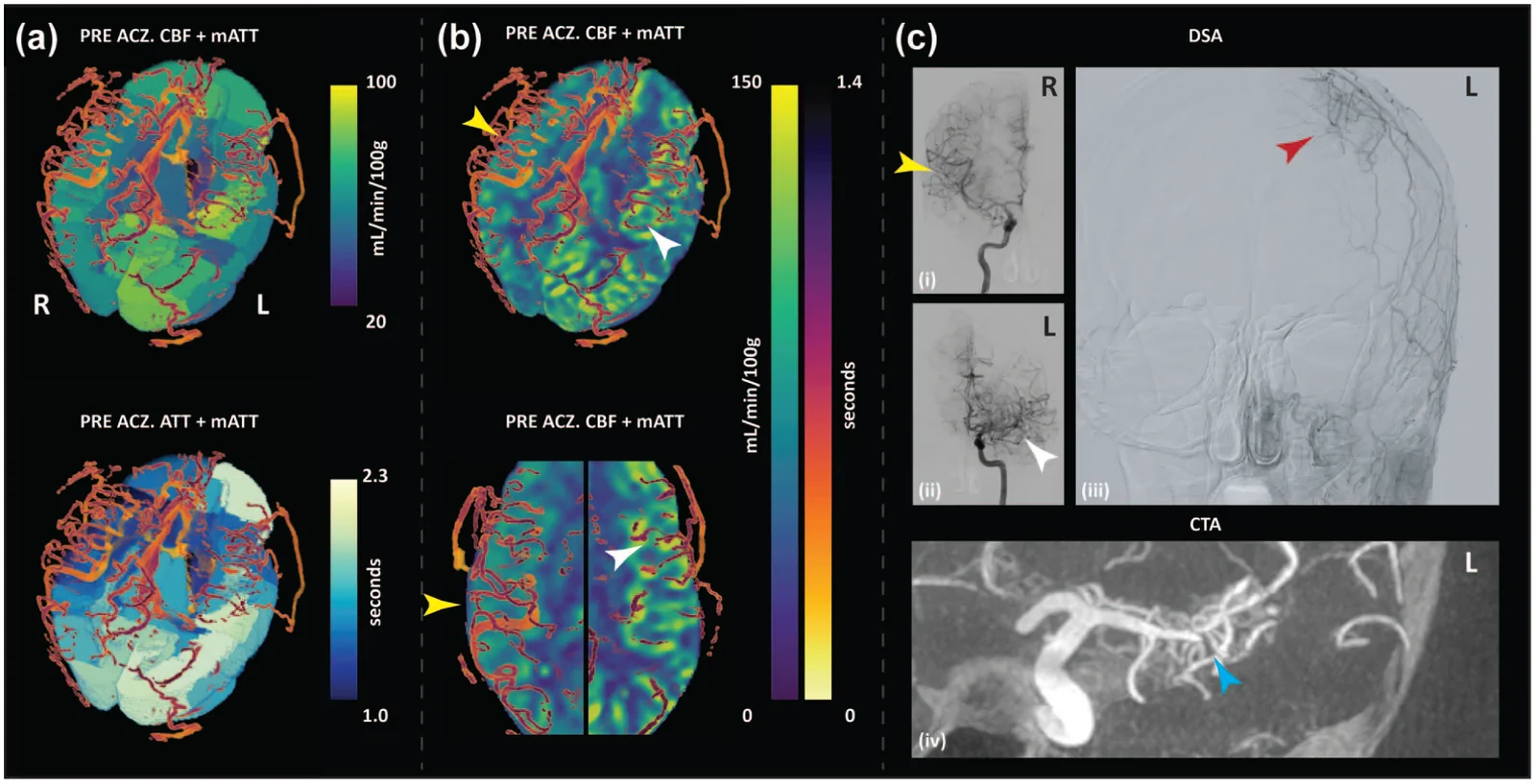
(a) Volume-rendered regional-average CBF and ATT maps overlaid with mATT (created via the SeeVieweR Toolbox^29^) demonstrate similar spatial patterns, with regions of elevated CBF corresponding to prolonged ATT; (b) Voxel-wise CBF maps show focal CBF hyperintensities that colocalize with large arteries exhibiting prolonged mATT, consistent with intravascular signal contamination (prolonged mATT: white arrows; unaffected CBF: yellow arrows); (c) These artifacts likely arise from slow flow within collateral vessels associated with the M1 stenosis (blue arrow) and the external–internal carotid bypass (red arrow). (m)ATT: (macrovascular) arterial transit time; CBF: cerebral blood flow; ACZ: acetazolamide; DSA: digital subtraction angiography; MIP: maximum intensity projection.

## Discussion

In this single-center study, we investigated the behavior of mATT and LBV derived from 4D-MRA during acetazolamide-induced vasodilation in participants with SOD. mATT demonstrated a strong correspondence with tissue-level ATT and shortened following acetazolamide administration, consistent with enhanced perfusion and preserved vasodilatory capacity. These findings support the physiological sensitivity of the artery-specific and regional level transit metrics and align with prior reports^7,30^ describing reduced transit times after pharmacologic vasodilation.

Despite these concordant vasodilatory responses, we did not observe the anticipated inverse relationship between transit-time changes and CVR. This likely reflects the complex hemodynamic effects of acetazolamide: while vasodilation reduces transit time within individual proximal vessels, it simultaneously promotes deeper downstream penetration of labeled blood into distal arterial territories.^13,31^ The inclusion of distal arterial voxels with intrinsically longer transit times can bias region averaged mATT estimates (Figure S1). This effect is specific to mATT, which directly samples arterial signal, and cannot be observed for tissue-level ATT. Accordingly, pre- and post-acetazolamide mATT curves converged at longer transit times, indicating that regional averaging influences macrovascular transit estimates under vasodilatory challenge.

Further attenuation of the relationships between (m)ATT and CVR likely reflects pathological hemodynamic processes inherent to steno-occlusive disease. These include vascular steal phenomena, arterial transit artifacts affecting CVR estimation, and collateral recruitment, which is directly captured by macrovascular imaging but may obscure simple associations with tissue-level reactivity measures. In contrast, LBV remained positively associated with CVR, suggesting that LBV may represent a more robust physiological marker of enhanced perfusion and cerebrovascular reserve during vasodilatory challenge. Compared with transit-based metrics, LBV may be less sensitive to regional averaging effects and may better reflect the net capacity for flow augmentation.

Across participants, mATT values were systematically shorter than ATT, consistent with the expected temporal delay between macrovascular inflow and tissue-level arrival and in agreement with prior work.^13^ Individual case examples further demonstrated concordant behavior of macrovascular (mATT and LBV) and tissue-level (CVR) metrics within defined regions of interest. Hemodynamically impaired regions exhibited negative CVR responses indicative of vascular steal, prolonged mATT, and reduced or absent ∆LBV following acetazolamide administration. In contrast, unaffected regions showed positive CVR, shortening of mATT, and increased LBV. This spatial concordance across modalities suggests that these parameters capture complementary manifestations of the same underlying hemodynamic state. The observed pattern likely reflects critically diminished inflow in compromised territories, such that labeled blood fails to adequately propagate into distal arterial segments within the imaging window. Notably, while LBV demonstrated robust detection of these flow deficits, the artery-specific nature of mATT provided enhanced spatial specificity for identifying pathological arterial segments. Collectively, these findings suggest that macrovascular 4D-MRA–derived metrics may provide greater specificity for detecting pathological arterial adaptations than tissue-level ATT alone, particularly in the presence of complex collateral flow patterns.

### Considerations

First, the modest sample size and the inherent heterogeneity associated with SOD meant that our statistical analyses were influenced by inter-individual variability. However, this also highlights that the 4D-MRA-derived parameters are sensitive to individual differences, which is of note considering that treatment decisions in this patient population are highly personalized. Nevertheless, larger studies in more homogeneous cohorts are warranted to evaluate the behavior of ΔmATT and ΔLBV across specific cerebrovascular pathologies and to establish their clinical relevance. While the present study was not designed to evaluate clinical outcomes, the observed hemodynamic findings may still have clinical implications for understanding hemodynamic compromise in SOD. Impaired CVR and steal responses reflect exhausted vasodilatory capacity and indicate possible (recurrent) stroke vulnerability.^32,33^ That said, at our medical center, hemodynamic imaging findings are not interpreted in isolation, as the multidisciplinary clinical decision making in patients with SOD integrates clinical symptoms, infarction patterns, angiographic disease severity, collateral circulation, and procedural considerations.^34^ Accordingly, future prospective studies combining artery-specific 4D-MRA parameters with clinical outcomes are required to determine whether these measures provide additional value for identifying hemodynamically vulnerable territories and improving patient selection for interventions such as revascularization.

Second, the 4D-MRA acquisition preceded multi-delay ASL, which may have reduced the apparent vasodilatory response in ∆mATT if acetazolamide had not reached its maximal effect; this likely further increased variability but was unavoidable considering the clinical nature of our acquisition protocol. Another potential explanation for the less pronounced acetazolamide-induced change in mATT is that mATT and ATT interrogate different stages of the vascular transit pathway. Although acetazolamide primarily reduces arteriolar resistance, thereby increasing cerebral blood flow throughout both the micro- and microvasculature,^35,36^ mATT reflects labeled-blood arrival within the proximal arterial tree, whereas ATT reflects transit to the tissue. Consequently, changes in mATT are not necessarily expected to mirror the magnitude of tissue-level ATT changes. Therefore, ATT and mATT should be considered complementary rather than interchangeable measures. For example, hemodynamic differences between macrovascular arterial transit and tissue-level perfusion responses could indicate the presence of collateral flow, identify regions with impaired downstream perfusion despite preserved arterial inflow, or provide insight into the site of hemodynamic impairment along the vascular pathway.

Looking forward, SOD-specific protocol optimizations can include more dense sampling of early inflow times to better characterize proximal collateral hemodynamics. Using the current 4DPACK implementation, such an approach would reduce scan time at the expense of distal vessel information, but would provide higher temporal resolution to support the mATT model fit.^37^ For clinical applications, reduced scan-time is preferable due to the sensitivity to motion artifacts (since this is a subtraction based technique). Alternatively, per-volume scan time could be decrease by using more aggressive under-sampling techniques such as compressed SENSE with AI-based denoising.^37^ Here it may be feasible to reduce overall scan-time or more densely sample the entire inflow phase (e.g. 0–2500 ms) for better model approximation. Future work should investigate the influence of inevitable SNR reduction on mATT model performance, and also which optimization pathways best align with disease characteristics.

### Conclusion

4D-MRA–derived metrics enable artery-specific assessment of cerebrovascular hemodynamics in steno-occlusive disease. While the relationship between CVR and ∆mATT may be influenced by collateral flow patterns, vascular steal, and transit-related effects, changes in LBV demonstrated a consistent positive association with CVR, indicating that ∆LBV may serve as a robust marker of arterial vasodilatory capacity. Together, these findings suggest that combined evaluation of ∆mATT and ∆LBV can provide additional insight into upstream arterial hemodynamics during vasodilatory challenge.

## Supplemental Material

sj-docx-1-jcb-10.1177_0271678X261500385 – Supplemental material for Quantification of arterial hemodynamics in steno-occlusive disease using time-resolved MRI-based angiography Supplemental material, sj-docx-1-jcb-10.1177_0271678X261500385 for Quantification of arterial hemodynamics in steno-occlusive disease using time-resolved MRI-based angiography by Quinten Deckers, Simone M Uniken Venema, Kees P Braun, Albert van der Zwan, Tristan PC van Doormaal, Pieter T Deckers, Jeroen CW Siero and Alex A Bhogal in Journal of Cerebral Blood Flow & Metabolism

## Data Availability

Data generated or analyzed during the study are available from the corresponding author by request, as stated in the written informed consents of the participants.*
