# Supplementary material for: Quantification of arterial hemodynamics in steno-occlusive disease using time-resolved MRI-based angiography
Source: J Cereb Blood Flow Metab. 2026 Oct 20:0271678X261500385. Online ahead of print. doi: 10.1177/0271678X261500385 (PMC13769707; doi:10.1177/0271678X261500385)
Supplement: Supplementary file 1 [file sj-docx-1-jcb-10.1177_0271678X261500385.docx]

**­­Supplementary material**

***Table S1:*** *Additional model information.*

*Prior to each linear mixed-effects model analysis, model selection was performed based on the Akaike Information Criterion (AIC), with the model demonstrating the lowest AIC value being selected as the optimal model. For all three measurements, model M6 demonstrated the lowest AIC and was therefore selected for subsequent analyses.*

**(m)ATT = (macrovascular) arterial transit time, CVR = cerebrovascular reactivity.*

| Model | Formula | Random effects |
| --- | --- | --- |
| M1 | CVR ~ ∆(m)ATT | (1 \| Subject) = random intercepts subjects for some subject variability |
| M2 | CVR ~ ∆(m)ATT | (∆(m)ATT \| Subject) = random slopes for subject variability |
| M3 | CVR ~ ∆(m)ATT | (1 \| Subject) + (1 \| Region) = random intercept subjects and regions for some variability in both |
| M4 | CVR ~ ∆(m)ATT | (∆(m)ATT \| Subject) + (1 \| Region) = random slopes for subject variability and random intercepts for some region variability |
| M5 | CVR ~ ∆(m)ATT | (1 \| Subject) + (∆(m)ATT \| Region) = random slopes for region variability and random intercepts for some subject variability |
| M6 | CVR ~ ∆(m)ATT | (∆(m)ATT \| Subject) + (∆(m)ATT \| Region) = random intercepts and slopes for subject and region variability. |

***Figure S1:*** *Simulation illustrating why mean arterial transit time (mATT) does not necessarily show a negative association with cerebrovascular reactivity (CVR), despite evidence of increased penetration depth.
(A) mATT angiogram of participant 3, highlighting an unaffected region selected for detailed inspection.
(B) Arterial label propagation in this region before and after acetazolamide (ACZ) administration. With an identical acquisition time window, post-ACZ conditions show visibly increased penetration depth (white arrow), consistent with enhanced flow.
(C) Conceptual illustration demonstrating how regional averaging attenuates the apparent mATT reduction. The blue curve represents pre-ACZ arterial label propagation, while the red curve represents post-ACZ propagation, characterized by faster and deeper penetration. At the penetration depth corresponding to pre-ACZ acquisition limit, the true mATT reduction is 0.80 s. However, when mATT values are averaged across the region up to the end of the acquisition window (≤ 2.2 s), the observed difference is reduced to only 0.22 s. This illustrates how spatial averaging can mask local transit-time shortening, explaining why mATT may fail to decrease despite increased CVR. Together with collateral recruitment, arterial transit artifacts, and vascular steal, these effects highlight the complexity of the relationship between CVR and mATT.*

**(m)ATT = (macrovascular) arterial transit time, CVR = cerebrovascular reactivity, LBV = labelled blood volume, ACZ = acetazolamide, AUC = area under the curve.*

***Figure S2:*** *Subject-specific relationships derived from linear mixed-effects models between cerebrovascular reactivity (CVR) and baseline arterial transit metrics. A) CVR versus baseline macrovascular arterial transit time (mATT) measured with 4D-MRA (p = 0.91; β = 0.32 ± 2.8).
B) CVR versus baseline arterial transit time (ATT) measured with arterial spin labeling (p = 0.27; β = −3.3 ± 2.9). Each data point represents a region-averaged value from an individual participant and is color-coded by participant.*

**(m)ATT = (macrovascular) arterial transit time, CVR = cerebrovascular reactivity, 4D-MRA = 4-dimensional MR-Angiography.*

***Figure S3:*** *Subject-specific relationships derived from linear mixed-effects models in unilateral steno-occlusive disease. Analyses were restricted to unilateral cases, with regions classified as affected or unaffected. A–D) Associations between cerebrovascular reactivity (CVR) and transit metrics are shown for affected and unaffected regions: A) affected CVR versus ΔATT, B) unaffected CVR versus ΔATT, C) affected CVR versus ΔmATT, and D) unaffected CVR versus ΔmATT. In unaffected regions, a significant interaction between ΔmATT and ΔLBV was observed (p = 0.007; β = 0.011 ± 0.004), indicating that the relationship between ΔmATT and CVR becomes more positive with increasing labeled blood volume. This interaction was not present in affected regions and was not observed for CVR–ΔATT relationships.
Also, a significant positive association between CVR and ΔATT was observed in unaffected regions (p = 0.002; β = 48.5 ± 7.8).*

**(m)ATT = (macrovascular) arterial transit time, CVR = cerebrovascular reactivity, LBV = labelled blood volume.*
